# Thrombectomy Outcomes for Anterior Circulation Stroke in the 6–24 h Time Window Solely Based On NCCT and CTA: A Single Center Study

**DOI:** 10.1007/s00062-024-01462-8

**Published:** 2024-10-01

**Authors:** Dmytro Shchehlov, Stanislav Konotopchk, Valentyna Pankiv, Farida Rzayeva, Sergii Kolomiichenko, Mykola Vyval, Fabian Flottmann, Jens Fiehler, Anna A. Kyselyova

**Affiliations:** 1 Scientific-practical Center of Endovascular Neuroradiology the NAMS of Ukraine, Kyiv, Ukraine; 2 Department of Diagnostic and Interventional Neuroradiology, University Medical Center Hamburg-Eppendorf, Martinistraße 52, 20246 Hamburg, Germany; 3 Clinic of Neuroradiology, University Hospital Zurich, Zurich, Switzerland

**Keywords:** Stroke, Thrombectomy, Late time window stroke

## Abstract

**Purpose:**

Since perfusion imaging may be unavailable in smaller hospitals, alternative imaging selection methods for acute ischemic stroke can improve outcomes and optimize resources. This study assessed the safety and effectiveness of using imaging criteria other than DEFUSE 3 and DAWN for thrombectomy beyond 6 h from symptom onset in patients stroke in the anterior circulation.

**Methods:**

This is a retrospective, single-center analysis of consecutive patients with large vessel occlusion in the anterior circulation undergoing thrombectomy. Patients were categorized into two groups based on the collateral status (moderate collaterals and good collaterals).

**Results:**

Among 198 patients, 106 (54%) met the inclusion criteria and were analyzed. Good collateral status was observed in 78 (74%) patients. Patients with good collaterals showed significantly lower mRS scores at discharge and at 90 days compared to their counterparts with moderate collateral status (4 (3–4) vs. 4 (4–5); *p* = 0.001 and 2 (0–4) vs. 6 (3–6); *p* < 0.001, respectively). More patients with good collateral status achieved favorable outcomes at 90 days compared to those with moderate status (48 (61.5%) vs. 5 (17.9%); *p* < 0.001). Good collaterals were an independent predictor of good clinical outcomes at 90 days (OR = 1.31, 95% CI: 1.13–1.53, *p* < 0.001).

**Conclusion:**

Selecting patients for endovascular treatment of acute ischemic stroke using non-contrast CT and CT angiography shows 90-day outcomes similar to the DAWN and DEFUSE-3 trials. Using collateral status on CT angiography can predict favorable outcomes after mechanical thrombectomy in resource-limited settings where perfusion imaging is unavailable.

## Introduction

Endovascular therapy has established itself as a state of the art strategy for patients with ischemic stroke due to a large vessel occlusion, also in a late-window stroke [1–6]. Time is one of the most critical factors in the treatment of acute ischemic stroke. Meta-analyses, incorporating data from these clinical trials, have shown that the likelihood of achieving functional independence after a stroke decreases with each hour of delay in endovascular reperfusion [7, 8]. This underscores the time-dependency and dynamic nature of stroke. However, uncertainties remain about the benefit and risk of endovascular intervention when undertaken more than 6 h after symptom onset as well as the degree to which benefit varies with time within the first 6 h after symptom onset. In 2018, the DEFUSE 3 and DAWN studies demonstrated the effectiveness of mechanical thrombectomy in patients receiving treatment beyond 6 h after the onset of stroke symptoms, using clinical presentation and perfusion imaging for inclusion in the study [9, 10]. Both studies showed the advantages in clinical outcome after endovascular treatment within 6–16 and 6–24 h from symptom onset. The specific timeframe at which recanalization therapies become ineffective varies among individuals. Consequently, during the extended treatment window, it is crucial to recognize and address individuals who may still derive potential benefits from endovascular interventions.

The AHA/ASA guidelines for extending the thrombectomy treatment window suggest that eligibility for endovascular therapy can be determined using either the DAWN or DEFUSE 3 trial criteria [11]. Although these randomized clinical trials are considered the gold standard of clinical evidence, the applicability of their results in clinical practice may be limited by excessively narrow inclusion criteria. Taking into consideration, that perfusion imaging might not be accessible in smaller hospitals, especially in the developing countries, using alternative clinical and imaging patient selection options could have a great impact on improving outcomes and optimizing resource utilization. Additionally, considering the risks of increased radiation, contrast-induced kidney injury, and longer treatment times, it is crucial to identify and validate simpler imaging-based selection criteria. Selection paradigms based on ASPECTS for late-presenting and wake-up stroke patients undergoing endovascular treatment demonstrate similar patient eligibility rates and comparable 90-day functional outcomes to those using DAWN’s clinical-core mismatch and DEFUSE-3’s perfusion-imaging mismatch criteria [12]. This approach allows for effective patient management and treatment planning, even in facilities with limited access to advanced imaging technologies.

Recent studies on late-presenting stroke with large vessel occlusion have demonstrated no significant differences in clinical outcomes between patients selected for recanalization using non-contrast computed tomography compared to those selected using CT Perfusion or MRI (CLEAR). Additionally, the MR CLEAN-LATE trial indicated that patient selection for endovascular treatment in the extended time window can be primarily based on the assessment of collateral flow using CT angiography [13, 14].

The aim of this study was to assess the safety and effectiveness of imaging criteria other than those in DEFUSE 3 and DAWN for mechanical thrombectomy beyond 6 h from symptom onset in patients with acute ischemic stroke due to a large vessel occlusion in the anterior circulation. We hypothesized that the presence of good collateral circulation is associated with favorable clinical outcomes in late-window stroke patients. Therefore, utilizing collateral status assessment could serve as a valuable tool in resource-limited settings.

## Methods

This is a retrospective, single-center analysis of consecutive patients with large vessel occlusion in the anterior circulation undergoing mechanical thrombectomy.

### Patient Selection

Patients undergoing endovascular treatment in the anterior circulation with a large vessel occlusion (internal carotid artery and middle cerebral artery) between January 2021 and January 2023 were included in the study.

All patients underwent multimodal stroke imaging (either directly at the Center or at the referring medical institutions)—non-contrast computed tomography (CT), single-phase CT angiography of the extracranial and intracranial arteries and in very few cases a perfusion imaging.

Patients meeting the following inclusion criteria were selected: (1) age >18 years, (2) functional independence before the stroke (mRS ≤ 2), (3) groin puncture performed between 6 and 24 h from the onset of first stroke symptoms, (4) complete periprocedural and data sets available, (5) collateral status ≥ 2 (moderate and good collateral status), (6) ASPECTS > 4. Perfusion imaging was not utilized in the study as the selection of the patients for the treatment was based on evaluating non-contrast CT data and collateral compensation assessed by CT angiography.

### Data Acquisition and Analysis

Data sets were retrospectively extracted from an institutional review board-approved database and clinical charts. Neuroradiological procedural data acquisition was performed by treating physicians and entered in a standardized form immediately after the endovascular procedure.

Data assessed included patients’ age, admission National Institutes of Health Stroke Scale (NIHSS) score, modified Rankin Scale score before admission, at discharge and at 90 days, time between symptom onset/last seen well to groin puncture and flow restoration and administration of intravenous thrombolysis.

Imaging assessment was performed by a trained neuroradiologist. Assessment of the initial and follow-up ischemic changes was conducted using the Alberta Score Program Early CT Score (ASPECTS and FU-ASPECTS) scale [15]. Collateral status was evaluated using CT-Angiography as described by Souza et al.: 0‑absent collaterals in > 50% of an MCA-M2 branch (superior or inferior division) territory; 1‑diminished collaterals in > 50% of an MCA-M2 branch territory; 2‑diminished collaterals in < 50% of an MCA-M2 branch territory; 3‑collaterals equal to the contralateral hemisphere; and 4‑increased collaterals [16]. A collateral status of 0 and 1 was classified as poor, while 2 was designated as moderate, and 3 and 4 were characterized as indicating good collateral flow.

Successful recanalization was defined as a eTICI score ≥ 2b [17]. The modified Rankin Scale (mRS) scores were used to assess functional outcomes, ranging from 0 (no disability) to 6 (death) and mRS ≤ 2 was considered a good outcome [18].

### Statistical Analysis

Statistical analyses were conducted using R software version 4.2.2. Patients were categorized into two groups based on the collateral status. To assess the statistical significance of the difference between compared groups based on frequency characteristics, the Chi-square test was used. Univariate associations were evaluated using the Mann–Whitney test (non-normally distributed data) or (normally distributed data) with a prior assessment of the distribution nature of primary data using the Shapiro-Wilk test. Logistic regression analysis was subsequently employed, with mRS at 90 days serving as the dependent variable. A significance threshold of *p* < 0.05 was applied, confidence intervals were set at 95%.

## Results

Among a total of 198 patients identified, recanalization procedures were not undertaken in 65 cases (33%) due to extensive infarction (ASPECTS < 4) and/or insufficient collateral circulation. Additionally, complete periprocedural or follow-up imaging and clinical data sets were unavailable for 27 patients (13%). 106 (54%) patients met the criteria and were included in the analysis. Good collateral status was observed in 78 (74%) patients.

**Fig. 1:**
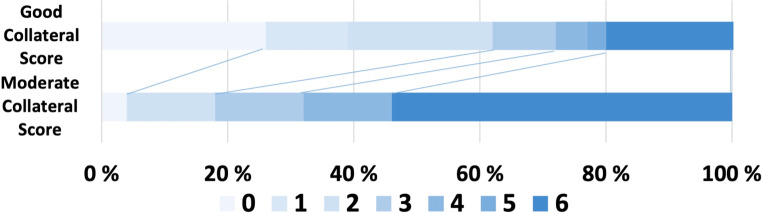
Distribution of mRS scores at 90 days after endovascular stroke treatment. *mRS* modified Rankin scale

In Table 1, our examination of baseline variables and clinical/periprocedural characteristics revealed an association between good collateral circulation, identified using CT angiography, and better functional outcomes both at discharge and 90 days post-stroke. Patients with good collateral status exhibited significantly lower mRS scores compared to their counterparts with moderate collateral status (4 (3–4) vs. 4 (4–5); *p* = 0.001 and 2 (0–4) vs. 6 (3–6); *p* < 0.001, respectively). Moreover, a significantly larger proportion of patients in the good collateral status group achieved favorable outcomes (mRS ≤ 2) in contrast to those in the moderate collateral status group (48 (61.5%) vs. 5 (17.9%); *p* < 0.001, Fig. 1).

**Table 1:** Characteristics of baseline variables, clinical and periprocedural characteristics tratified by collateral status

Variable	Good collateral status, *n* = 78 (74%)	Moderate collateral status, *n* = 28 (26%)	*p*-Value
Age, median (IQR)	68 (61–73)	71 (65–76.2)	0.150
Female, *n* (%)	43 (55.1%)	13 (46.4%)	0.568
*Occlusion location:*			
Tandem, *n* (%)	19 (24.4%)	9 (32.1%)	0.581
ICA, *n* (%)	29 (37.2%)	12 (42.9%)	0.762
MCA-M1, *n* (%)	49 (62.8%)	16 (57.1%)	0.198
Collateral score, median (IQR)	3 (3–3)	2 (2–2)	*<* *0.001*
*Stroke Type:*			0.808
Cardioembolic, *n* (%)	54 (66.7%)	22 (75.0%)	–
Cryptogenic, *n* (%)	8 (10.3%)	1 (3.57%)	–
Dissection, *n* (%)	1 (1.28%)	0 (0%)	–
Pre-existing stenosis, *n* (%)	15 (19.2%)	5 (17.9%)	–
ASPECTS, median (IQR)	9 (8–10)	8 (6–9)	*0.004*
NIHSS at admission, median (IQR)	15 (11–18)	16 (15–20)	*0.017*
Iv.thrombolysis, *n* (%)	24 (30.8%)	7 (25.0%)	0.739
Time from symptom onset to groin puncture, min (SD)	521 (171)	642 (315)	0.061
Time from symptom onset to recanalization, min (SD)	581 (181)	694 (308)	0.075
Number of recanalization passes	2 (1–3)	3 (2–4)	0.180
*First pass technic:*			0.639
ADAPT, *n* (%)	47 (60.3%)	15 (53.6%)	–
Combined method (SAVE or Solumbra), *n* (%)	30 (38.5%)	13 (46.4%)	–
Stentretriever only, *n* (%)	1 (1.28%)	0 (0%)	–
*eTICI Score*			0.150
0, *n* (%)	10 (13%)	3 (11%)	–
1, *n* (%)	0 (0%)	1 (4%)	–
2а, *n* (%)	0 (0%)	2 (7%)	–
2b, *n* (%)	10 (13%)	4 (14%)	**–**
2c, *n* (%)	19 (24%)	7 (25%)	**0.07**
3, *n* (%)	39 (50%)	11 (39%)	–
FU-ASPECTS at 24 h, median (IQR)	6 (5–7)	3 (2–5)	*<* *0.001*
mRs at discharge, median (IQR)	4 (3–4)	4 (4–5)	*0.001*
mRs at 90 days, median (IQR)	2 (0–4)	6 (3–6)	*<* *0.001*
Good outcome (mRS ≤ 2), *n* (%)	48 (62%)	5 (18%)	*<* *0.001*

*IQR* interquartile range, *SD* standard deviation, *ICA* Internal Carotid Artery, *MCA-M1* Middle Cerebral Artery-M1 Segment, *ASPECTS* Alberta Stroke Program Early CT Score, *NIHSS* National Institutes of Health Stroke Scale, *Iv.thrombolysis* Intravenous Thrombolysis, *ADAPT* A Direct Aspiration First Pass Technique, *SAVE* Suction and Aspiration of Vessels Distal to Clot Technique, *eTICI* Extended Thrombolysis in Cerebral Infarction, *mRs* Modified Rankin Scale, *FU-ASPECTS* Follow-Up Alberta Stroke Programme Early CT Score

Furthermore, patients with good collateral status had a significantly higher median Alberta Stroke Program Early CT Score (ASPECTS) at admission compared to those with moderate collateral status (9 (8–10) vs. 8 (6–9); *p* = 0.004). Additionally, the National Institutes of Health Stroke Scale (NIHSS) scores at admission were significantly lower in patients with favorable collateral status (15 (11–18) vs. 16 (15–20); *p* = 0.017).

Periinterventional complications were recorded in 13 (12.3%) cases, including embolization to new arterial territories in 10 (9.4%) patients, dissection of the internal carotid artery in 1 (0.9%) patient, and intracerebral hemorrhage due to artery perforation/rupture in 2 (1.9%) cases. 80% of the patients were transferred from other centers for the endovascular treatment.

Successful recanalization was achieved in a higher percentage of patients with good collaterals, than in patients with moderate collaterals (87% vs. 78%; *p* = 0.07).

Logistic regression analysis was performed with good clinical outcome (mRS ≤ 2) as the dependent variable. Good collateral status emerged as an independent predictor for good clinical outcome at 90 days with an adjusted odds ratio of 1.31 (95% CI: 1.13–1.53, *p* < 0.001) (Table 2; Fig. 1).

**Table 2:** Logistic regression analysis of predictors for functional outcome

Variable	Odds Ratio	95% CI	*p-*Value
Collateral score	1.31	1.13–1.53	*<* *0.001*
Time from symptom onset to groin puncture, min (SD)	1.00	1.00–1.00	0.84
NIHSS at admission	0.99	0.97–1.00	0.11
ASPECTS	1.04	0.98–1.11	0.16

*ASPECTS* Alberta Stroke Program Early CT Score, *NIHSS* National Institutes of Health Stroke Scale

## Discussion

In this retrospective study, we utilized the imaging criteria other than those in DEFUSE 3 and DAWN for mechanical thrombectomy beyond 6 h from symptom onset in patients with acute ischemic stroke due to a large vessel occlusion in the anterior circulation. We observed, that patients who presented with a stroke in the anterior circulation beyond 6 h from symptom onset, ASPECTS > 4 and had good collateral status on the CTA had significantly better functional outcomes both at discharge and 90 days post-stroke, compared to patients with moderate collaterals. Although recanalization was successful (TICI 2b-3) in 22 patients (79%) with moderate collateral status, only 5 of these patients (8%) experienced a good clinical outcome at 90 days, with a median mRS of 6 (IQR: 3–6).

The groundbreaking randomized trials DEFUSE‑3 and DAWN have demonstrated that the therapeutic window for endovascular treatment in patients with acute ischemic stroke due to large vessel occlusion can be extended up to 24 h in certain individuals based on advanced imaging and clinical assessment data. Both studies demonstrated the effectiveness of endovascular treatment within 6–16 (DEFUSE-3) and 6–24 (DAWN) hours after symptom onset. Good clinical outcomes at 90 days were achieved in 45% and 49%, respectively [9, 10]. Pizzo et al. has also shown economical effectiveness of mechanical thrombectomy between 6 to 24 h after symptom onset: despite higher initial stroke treatment costs, it results in reduced costs in the stroke care pathway due to better outcomes [19]. Our results align with those of these two trials. The rate of successful revascularization was slightly higher in our study (84.9%, compared to 84% in DAWN and 76% in DEFUSE-3), as was the functional independence rate at 90 days (mRS ≤ 2) (61.5%, compared to 49% in DAWN and 45% in DEFUSE-3). While selecting patients based on optimal and strict CT perfusion parameters may enhance clinical outcomes, this approach risks excluding numerous patients who could have benefited from endovascular treatment, particularly in centers where perfusion imaging is not readily available [20, 21].

In our series, some patients would not have qualified for endovascular treatment according to the inclusion criteria of the DAWN and DEFUSE-3 trials: e.g. mismatch between core and NIHSS (DAWN), although they had good collaterals and/or CT findings suggestive of thrombectomy eligibility. Some patients deviated from the DEFUSE 3 criteria, but their collateral status appeared promising.

One limitation of the CLEAR study is its lack of assessment of collateral status, which our study specifically evaluated [14]. Our findings are also comparable with the outcomes of the MR CLEAN-LATE trial, which suggested that the selection of patients for endovascular treatment in the late window may predominantly rely on the presence of collateral flow [13]. Among patients with well-developed collaterals, a good functional outcome at 90 days was observed in 61.5% of cases, while in the case of poor collateral compensation, it was noted in only 17.9%.

Our data align as well with the previously published data, that suggests that endovascular treatment can be effective and safe in patients with ischemic stroke > 6 h from onset selected without CT perfusion or magnetic resonance imaging or relying just on the non-contrast CT [14, 22]. Two factors may explain this equivalence of treatment outcomes in patients selected based on data from different visualization methods.

Firstly, previous studies have demonstrated a moderate correlation between the affected area by ASPECTS on non-contrast CT and the infarct core volume by CT perfusion data [23–25], but this correlation was stronger in patients with large vessel occlusion and longer delay from stroke onset, so that ASPECTS could be used as a surrogate marker of CT perfusion infarct core in patients with late presenting stroke. This result was highly robust, as the association persisted even after adjusting for multiple clinical and radiological factors. Additionally, an independent relationship between higher ASPECTS scores and good collateral status was identified, reinforcing the importance of collateral circulation in preventing early tissue loss [25].

Secondly, among patients with the same ASPECTS, clinical core mismatch does not significantly decline over time when presenting in the late time window, with ASPECTS score and not time being an independent predictor of this mismatch [26]. These results support the usage of ASPECTS-based paradigm in late window stroke. Utilizing this paradigm in the late window stroke has been shown to have 90-day outcomes comparable to those in the DAWN and DEFUSE-3 studies [12]. In line with this Santos et al. demonstrated that thrombectomy can be safe and effective in patients with wake-up stroke and late-window stroke selected based on the clinical-core mismatch [27].

A higher percentage of successful recanalization was observed in patients with good collaterals compared to those with moderate collaterals. This could be explained through the impaction force, which is influenced by the pressure gradient across the thrombus. Lower systemic blood pressure acting on the proximal thrombus, combined with enhanced retrograde collateral flow, reduces the pressure gradient across the occluding thrombus, thereby increasing the likelihood of successful recanalization [28]. This highlights another advantage of incorporating collateral assessment, along with ASPECTS and NIHSS, as shown in previous studies, when selecting patients for endovascular treatment in cases of late-window stroke.

Our data sets included single-phase CTA, though current evidence indicates that using multiphase CTA during the initial evaluation of acute stroke patients can offer important clinical benefits, such as more accurate detection of large-vessel occlusions, greater resilience to issues like patient movement, as well as it offers higher consistency between evaluators. However known multi-phase CTA limitations like late opacification of pial vessels due to stenosis in the proximal vessels, poor hemodynamic situation and posterior circulation demonstrate that single-phase CTA retains its validity and remains a useful tool in stroke assessment, especially in cases where multi-phase CTA may not provide optimal results or is unavailable [29].

Taking into consideration, that perfusion imaging or multi-phase CT angiography might not be accessible in smaller hospitals especially in the developing countries, using alternative clinical and imaging patient selection options could have a great impact on improving outcomes and optimizing resource utilization.

### Limitations

One of the limitations of our study is the presence of multiple confounding variables that may affect the validity of our findings. Specifically, the cohort with a good collateral score presented with lower NIHSS scores at admission, higher ASPECTS, fewer thrombectomy passes, compared to the patients with moderate collaterals. These differences suggest that patients with better collateral status had more favorable initial conditions, also due to their good collaterals. One of the inclusion criteria for the study was a pre-stroke mRS score of 0‑2. Unfortunately, no detailed mRS data for each patient are available, so they could not be assessed in the statistical analysis. Higher mRS score (> 3) was one of the factors that contributed to the decision not to treat certain patients in the first place. Additionally, complete periprocedural or follow-up imaging and clinical data sets were unavailable for 27 patients (13%) who received thrombectomy, so we had to exclude them from our analysis. Our study results are derived from retrospective analysis, which has well-known limitations—most importantly, the potential forselection bias. The sample size is limited, especially in the group of patients with moderate collaterals. Availability of only single-phase CT angiography is also a limiting facctor in the evaluation of collateral status. Despite these limitations, the study highlights the potential utility of using non-contrast CT and CT angiography for patient selection in resource-limited settings.

## Conclusion

Since perfusion imaging may not be available in smaller or resource-limited medical facilities, using different methods for selecting patients for endovascular treatment for acute ischemic stroke in the anterior circulation beyond 6 h after symptom onset are essential. Therefore, we believe that good collateral status can be a valuable additional patient selection tool in such scenarios and a predictor of favorable clinical outcome.

## Data Availability

N/A
